# Support for Indoor Bans on Electronic Cigarettes among Current and Former Smokers

**DOI:** 10.3390/ijerph111212174

**Published:** 2014-11-25

**Authors:** Stephanie K. Kolar, Brooke G. Rogers, Monica Webb Hooper

**Affiliations:** 1 Department of Psychology, College of Arts and Sciences, University of Miami, Coral Gables, FL 33146, USA; E-Mails: skolar@miami.edu (S.K.K.); brogers@psy.miami.edu (B.G.R.); 2 Sylvester Comprehensive Cancer Center, Miami, FL 33136, USA

**Keywords:** electronic cigarette, E-cigarette, vaping, secondhand vapor, nicotine, environmental smoke

## Abstract

Objectives: Electronic cigarette (e-cigarette) use is increasing in the U.S. Although marketed as a safer alternative for cigarettes, initial evidence suggests that e-cigarettes may pose a secondhand exposure risk. The current study explored the prevalence and correlates of support for e-cigarette bans. Methods: A sample of 265 current/former smokers completed a cross-sectional telephone survey from June–September 2014; 45% Black, 31% White, 21% Hispanic. Items assessed support for home and workplace bans for cigarettes and e-cigarettes and associated risk perceptions. Results: Most participants were aware of e-cigarettes (99%). Results demonstrated less support for complete e-cigarette bans in homes and workplaces compared to cigarettes. Support for complete e-cigarette bans was strongest among older, higher income, married respondents, and former smokers. Complete e-cigarette bans were most strongly endorsed when perceptions of addictiveness and health risks were high. While both e-cigarette lifetime and never-users strongly supported cigarette smoking bans, endorsement for e-cigarette bans varied by lifetime use and intentions to use e-cigarettes. Conclusions: Support for indoor e-cigarette bans is relatively low among individuals with a smoking history. Support for e-cigarette bans may change as evidence regarding their use emerges. These findings have implications for public health policy.

## 1. Introduction

The largest advances in tobacco control have come from legislative policies to enforce public smoking bans. In the U.S., support for smoking restrictions originated from grassroots efforts among nonsmokers who formed groups such as Americans for Nonsmokers’ Rights (ANR). Since the initiation of the nonsmokers’ rights movement in 1976, over 4063 municipalities have enacted laws restricting smoking in public places. In 39 states and territories, 100% smoke-free laws exist for workplaces and/or restaurants and bars [1]. Similar movements have taken place internationally, with smoking bans currently enforced in Canada, Australia, Hong Kong, and others [2].

The public health impact of bans on smoking is significant. In several epidemiological studies, the number of hospital admissions due to acute myocardial infarctions decreased significantly following the effecting of city-wide smoking bans [3,4,5]. Meta-analytic findings have also demonstrated inverse associations between smoking ban implementation and hospital admission rates for other cardiovascular, respiratory, and cerebrovascular conditions [6]. Smoking bans are the most effective method for reducing environmental tobacco smoke, with estimates ranging from a 71%–100% reduction in exposure following implementation of a ban [7]. Living or working in environments with complete or partial (smoking is restricted to designated areas) bans is associated with smoking reductions, increased quit attempts, and/or smoking cessation [8]. Compared to workplaces with flexible or no bans, complete workplace bans have been found to reduce smoking prevalence by 6% and smoking intensity by 14% [9].

Public support for smoking bans is important for the enactment of legislation restricting smoking. Moreover, once bans are implemented, support for restrictions also increases among current smokers [10,11,12]. Although a greater proportion of non-smokers support indoor and outdoor smoking bans [10,13,14,15], some recent evidence indicates that the majority of smokers support indoor bans [16]. Moreover, support for smoke-free laws is positively associated with quit attempts [17].

We located only one previous study that examined support for bans on electronic cigarettes or e-cigarettes. Wolfson *et al.* [18] found that 43% of respondents supported complete e-cigarette bans in restaurants. In addition, support for restaurant bans was lower among cigarette smokers and/or e-cigarette users, and younger, unmarried, and lower educated participants [18]. Understanding public views on e-cigarettes is important, as these products are gaining popularity in the U.S. [19]. Indeed, over 75% of adults report e-cigarette awareness [20,21] and nearly half of current or former smokers report lifetime e-cigarette use [22]. There is growing concern about the possible negative effects of both direct and environmental exposure. Scientific data examining the potential health consequences of e-cigarettes is emerging; however, there is initial evidence of potentially harmful compounds including aldehydes [23,24], ultrafine particles, and aerosolized nicotine [25]. When exhaled, vapor is dispersed into the environment and can be inhaled by other individuals in the form of secondhand vapor (SHV) or spread to surfaces or objects (*i.e.*, thirdhand vapor) [26,27].

Few regulations have been enacted that specifically restrict e-cigarette use in all public places, although some laws enacted before e-cigarettes were available may be interpreted to include e-cigarettes [28]. The current study assessed support for e-cigarette bans among smokers and former smokers who completed a telephone survey. Specifically, we compared support for indoor cigarette smoking* versus* e-cigarette use restrictions, and examined associations between support for e-cigarette bans and demographic characteristics, risk perceptions, lifetime e-cigarette use, and intentions to use e-cigarettes.

## 2. Methods

### 2.1. Participants and Data Collection

Participants were recruited through Internet advertisements, flyers, and community outreach from June to September 2014. Eligibility criteria included 18 years of age or older, ability to read and speak English, and current (defined as smoking at least 100 lifetime cigarettes and current smoking on at least some days) or former cigarette smoker (lifetime history of 100 cigarettes, but not currently smoking). Following verbal informed consent, participants completed a 20-min interviewer-administered survey. Participants received a $10 gift card for completing the survey. The University of Miami Institutional Review Board approved this study.

### 2.2. Measures

#### 2.2.1. Demographics and Smoking Status

Participants self-reported race/ethnicity, sex, age, marital status, annual household income, education, employment, health insurance status, sexual orientation, and current smoking status.

#### 2.2.2. E-Cigarette Awareness, Lifetime Use, and Behavioral Intentions

A single item assessed whether participants had ever heard of an electronic cigarette; those who were aware of electronic cigarettes were asked additional items on e-cigarettes. Single items assessed e-cigarette lifetime use with response options of “Yes” or “No” and intentions to use e-cigarettes in the next year with response options of “Very likely”, “Somewhat likely”, and “Not at all likely”.

#### 2.2.3. Risk Perceptions

We assessed risk perceptions for both e-cigarettes and cigarettes, with a focus on health risks and addictiveness. Two items were adapted from the 2009–2010 National Adult Tobacco survey (NATS) [29] to assess risk perceptions. Health risk perception (one item) assessed the extent to which breathing smoke or vapor from other people’s cigarettes or from other tobacco products was perceived to be “not at all bad for your health”, “somewhat bad for your health”, or “very bad for your health”, Perceived addictiveness of both cigarettes and e-cigarettes was assessed via single items with response options including “not at all addictive”, “somewhat addictive”, or “very addictive”.

#### 2.2.4. Indoor Smoking Bans

Four items adapted from the NATS assessed views on indoor use restrictions for e-cigarettes and cigarettes. Two items assessed support for cigarette and e-cigarette bans at homes and workplaces with response options including “always allowed”, “allowed at some times or in some places”, or “never allowed”. An additional two items assessed personal home bans for e-cigarettes and cigarettes. Participants employed outside of their home were also asked about smoking bans at their workplace using the aforementioned response options, in addition to options indicating no workplace policy or uncertainty about the policy.

### 2.3. Statistical Analysis

Chi-square and Fisher’s exact tests examined responses to the risk perceptions and indoor smoking restrictions items for e-cigarettes and cigarettes. We also compared responses to each of the smoking ban items by demographic factors, lifetime e-cigarette use, and intentions to use e-cigarettes. Selection of the “never allowed” response option was operationalized as support for a complete ban. *p*-Values less than 0.05 were considered statistically significant. Because this is the first study to examine views on e-cigarette bans, we considered our analyses exploratory and did not make adjustments for multiple comparisons. SAS version 9.3 was used to conduct all analyses.

## 3. Results

The survey was completed by 265 participants (89% of screened). Two percent (*n* = 7) were ineligible because they were not current or former smokers, 3% (*n* = 9) were not interested, 5% (*n* = 16) could not be reached by return telephone call to complete the survey, and <1% (*n* = 2) were eligible, but did not complete the survey. As shown in Table 1, 23% self-identified as Hispanic, 30% as non-Hispanic White, 43% as Black/African American, and 4% as another race or multiracial. The overall sample was mostly female, middle-aged (median = 44.0, IQR = 21.0), unmarried/single, lower income, heterosexual, and completed high school. Employment status was varied with over half employed full or part-time; 5% were students, 16% disabled, 4% retired, and 4% were homemakers. Most participants were current cigarette smokers.

**Table 1:** Demographic characteristics and support for indoor home and workplace e-cigarette bans.

	Total	Inside A Home, Smoking E-Cigarettes Should Be				At Workplaces, Smoking E-Cigarettes Should Be			
		Never Allowed	Allowed at Some Times or Places	Always Allowed		Never Allowed	Allowed at Some Times or Places	Always Allowed	
	(*n* = 265)	(*n* = 58)	(*n* = 94)	(*n* = 112)	*p*-Value	(*n* = 93)	(*n* = 108)	(*n* = 64)	*p*-Value
Racial/Ethnic group ^*^					0.25				0.17
White	30% (77)	24% (19)	34% (27)	42% (33)		38% (30)	46% (36)	16% (13)	
Black or African American	43% (115)	18% (21)	33% (38)	49% (56)		29% (33)	43% (50)	28% (32)	
Hispanic	23% (61)	26% (16)	43% (26)	31% (19)		41% (25)	33% (20)	26% (16)	
Other	4% (10)	22% (2)	33% (3)	45% (4)		50% (5)	20% (2)	30% (3)	
Gender					0.93				0.53
Female	60% (160)	21% (34)	37% (58)	42% (67)		37% (60)	41% (65)	22% (35)	
Male	40% (104)	23% (24)	35% (36)	42% (44)		32% (33)	41% (43)	27% (28)	
Age group					0.01				0.54
18 to 30	25% (66)	12% (8)	44% (29)	44% (29)		29% (19)	39% (26)	32% (21)	
31 to 45	29% (77)	26% (20)	31% (24)	43% (33)		38% (29)	40% (31)	22% (17)	
46 to 55	29% (76)	20% (15)	28% (21)	52% (40)		37% (28)	38% (29)	25% (19)	
>55	17% (46)	33% (15)	45% (20)	22% (10)		37% (17)	48% (22)	15% (7)	
Marital status					0.02				0.37
Unmarried/Single	58% (153)	18% (28)	43% (65)	39% (60)		33% (50)	44% (67)	23% (36)	
Married/Living with a partner	20% (53)	28% (15)	17% (9)	55% (29)		34% (18)	34% (18)	32% (17)	
Separated/Divorced/Widowed	22% (59)	26% (15)	34% (20)	40% (23)		42% (25)	39% (23)	19% (11)	
Sexual Orientation					0.55				0.29
Heterosexual	91% (242)	22% (53)	37% (88)	41% (100)		34% (82)	41% (99)	25% (61)	
Bisexual/Homosexual/Not sure	9% (23)	22% (5)	26% (6)	52% (12)		48% (11)	39% (9)	13% (3)	
Annual household income					0.048				0.03
Under $10,000	36% (94)	25% (24)	31% (29)	44% (41)		36% (34)	32% (30)	32% (30)	
$10,001–$20,000	17% (45)	16% (7)	51% (23)	33% (15)		38% (17)	53% (24)	9% (4)	
$21,001–$40,000	27% (69)	13% (9)	41% (28)	46% (31)		26% (18)	49% (34)	25% (17)	
$40,001 or more	20% (53)	32% (17)	26% (14)	42% (22)		41% (22)	36% (19)	23% (12)	
Education					0.39				0.32
Less than HS diploma	21% (54)	28% (15)	33% (18)	39% (21)		43% (23)	33% (18)	24% (13)	
HS diploma/GED	24% (64)	14% (9)	33% (21)	53% (33)		31% (20)	42% (27)	27% (17)	
Business/Technical training or some college	36% (96)	21% (20)	36% (35)	43% (41)		29% (28)	43% (41)	28% (27)	
College degree (2-yr/4-yr/graduate)	19% (51)	27% (14)	40% (20)	33% (17)		43% (22)	43% (22)	14% (7)	
Employment					0.51				0.34
Employed Full-time	26% (68)	26% (18)	37% (25)	37% (25)		37% (25)	41% (28)	22% (15)	
Unemployed, looking for work	12% (32)	12% (4)	47% (15)	41% (13)		28% (9)	59% (19)	13% (4)	
Employed part-time	32% (85)	21% (18)	37% (31)	42% (35)		33% (28)	39% (33)	28% (24)	
Student/Disabled/Retired/Homemaker	30% (79)	23% (18)	29% (23)	48% (38)		38% (30)	35% (28)	27% (21)	
Smoking status					0.07				0.049
Former smoker	21% (55)	29% (16)	42% (23)	29% (16)		49% (27)	33% (18)	18% (10)	
Current smoker	79% (210)	20% (42)	34% (71)	46% (96)		31% (66)	43% (90)	26% (54)	

^*^ *p*-value calculated does not include “Other” race. Abbreviations: GED = General Equivalency Diploma; HS = High School; IQR = Interquartile Range.

### 3.1. Support for Indoor Cigarette Smoking Versus E-Cigarette Use Restrictions

Results demonstrated significant differences in support for cigarette smoking and e-cigarette use bans (Table 2). With regard to home settings, almost two-thirds of participants supported complete cigarette smoking bans, with a minority endorsing no restrictions. In contrast, support for complete e-cigarette bans in homes was almost three times lower than cigarettes, with the majority endorsing no restrictions in homes. A similar pattern was observed for personal home restrictions, such that support for complete cigarette smoking bans was almost twice as strong compared to support for e-cigarette restrictions inside of their homes. Most participants endorsed no e-cigarette restrictions in their homes. In the workplace, support for complete cigarette smoking bans more than doubled support for complete e-cigarette bans, with the majority endorsing partial e-cigarette restrictions in work settings. Among participants employed outside of their home, reports of complete cigarette smoking bans at work were more than 2.5 times greater than e-cigarette workplace bans. Approximately one-half of the sample reported no workplace e-cigarette policy or uncertainty about related policy.

**Table 2:** Support for indoor smoking restrictions for e-cigarettes *versus* cigarettes.

Indoor Smoking Restrictions	Cigarettes	E-Cigarettes	*p*-Value
In your opinion, inside a home, smoking should be:			<0.01
Never allowed	63% (167)	22% (58)	
Allowed only at some times/places	23% (61)	36% (94)	
Always allowed	14% (36)	42% (112)	
Inside your home (not counting decks, porches, or garages), smoking is:			<0.01
Never allowed	63% (166)	32% (86)	
Allowed only at some times/places	15% (40)	23% (61)	
Always allowed	22% (59)	45% (118)	
At workplaces, do you think smoking indoors should be			<0.01
Never allowed	83% (219)	35% (93)	
Allowed only at some times/places	16% (43)	41% (108)	
Always allowed	1% (3)	24% (64)	
At your workplace (outside of your home), is smoking in indoor areas ^*^			<0.01
Never allowed	81% (81)	30% (30)	
Allowed only at some times/places	6% (6)	6% (6)	
Always allowed	2% (2)	15% (15)	
There is no policy at my work	8% (8)	24% (24)	
I’m not sure what the policy is	3% (3)	25% (25)	

^*^ Among participants who worked outside of the home (*n* = 100)

### 3.2. Associations between Support for Indoor E-Cigarette Restrictions and Demographic Characteristics

Support for indoor e-cigarette restrictions in homes differed by age, marital status, and household income (Table 1). Compared to the 18–30 year age group, older participants (>55 years) were more supportive of complete e-cigarette bans in homes. In contrast, participants in the youngest age category were more likely to endorse partial e-cigarette bans or no restrictions in home settings. Unmarried/single participants were the most supportive of partial e-cigarette bans in homes, compared to those who were no longer married. Married participants were least supportive of partial bans, preferring either no restrictions or complete bans, respectively. Participants who reported annual household income levels over $40,000 were most likely to support complete e-cigarette restrictions at home, compared to those reporting lower income. Compared to former smokers, current smokers were less likely to support complete or partial e-cigarette bans at home (*p* = 0.07).

Support for indoor e-cigarette bans in workplaces differed by income and smoking status. Specifically, participants who reported annual household income levels over $40,000 were most likely to support complete e-cigarette restrictions at work, compared to those reporting lower income. Compared to current smokers, former smokers were more supportive of complete e-cigarette bans in workplaces.

### 3.3. Associations between Support for E-Cigarette Bans and Risk Perceptions

Results demonstrated significant associations between support for indoor e-cigarette restrictions and perceptions of risk (Table 3). The strongest supporters of complete e-cigarette bans in home settings were participants who perceived e-cigarettes to be highly addictive. Correspondingly, partial bans were most supported when e-cigarettes were perceived as somewhat addictive, and no home restrictions were strongly endorsed when perceived as non-addictive. With regard to personal home restrictions, the same pattern was observed, such that complete e-cigarette bans were most likely when perceptions of addictiveness were highest, partial bans were most prevalent with moderate addictiveness perceptions, and no restrictions were more strongly endorsed when perceived as non-addictive. Finally, the same pattern was found for support for workplace e-cigarette bans. Support for complete bans was strongest when e-cigarettes were perceived to be highly addictive, followed by partial ban support with moderate addictiveness perceptions, and no support when e-cigarettes were perceived as non-addictive.

We also found significant associations between support for indoor e-cigarette restrictions and health risk perceptions. The strongest supporters of complete e-cigarette bans in home settings were participants who perceived e-cigarettes to confer significant health risks. Correspondingly, partial bans were most supported when e-cigarettes were perceived as somewhat harmful for health and no home restrictions were strongly endorsed when perceived as non-harmful. With regard to personal home restrictions, the same pattern was observed, such that complete e-cigarette bans were most likely when perceptions of health risk were highest, partial bans were most prevalent with moderate health risk perceptions, and no restrictions were more strongly endorsed when perceived as non-harmful. Lastly, the same pattern was found for support for workplace e-cigarette bans. Support for complete bans was strongest when e-cigarettes were perceived to confer a significant health risk, followed by partial ban support with moderate health risk perceptions, and no support when e-cigarettes were perceived as non-harmful.

**Table 3:** Associations between support for indoor e-cigarette bans and risk perceptions.

	Smoking E-Cigarettes Is				Breathing Vapor from Other People’s E-Cigarettes Is			
	Not Addictive	Somewhat Addictive	Very Addictive	*p*-Value	Not Bad for Health	Somewhat Bad	Very Bad for Health	*p*-Value
	(*n* = 57)	(*n* = 154)	(*n* = 52)		(*n* = 107)	(*n* = 123)	(*n* = 34)	
Inside a home smoking e-cigarettes should be				<0.01				<0.01
Never allowed	16% (9)	19% (29)	38% (20)		8% (9)	23% (28)	62% (21)	
Allowed at some times or places	23% (13)	44% (68)	23% (12)		23% (25)	51% (62)	21% (7)	
Always allowed	61% (35)	37% (56)	38% (20)		68% (73)	26% (32)	18% (6)	
At my home smoking e-cigarettes is				<0.01				<0.01
Never allowed	23% (13)	29% (45)	52% (27)		17% (18)	35% (43)	74% (25)	
Allowed at some times or places	21% (12)	28% (43)	12% (6)		18% (19)	29% (36)	18% (61)	
Always allowed	56% (32)	43% (66)	37% (19)		65% (70)	36% (44)	9% (3)	
At workplaces, smoking e-cigarettes should be				<0.01				<0.01
Never allowed	21% (12)	33% (51)	56% (29)		18% (19)	41% (50)	71% (24)	
Allowed at some times or places	30% (17)	50% (78)	25% (13)		42% (43)	45% (56)	18% (6)	
Always allowed	49% (28)	16% (25)	19% (10)		40% (43)	14% (17)	12% (4)	

**Table 4:** Associations between support for indoor smoking bans, lifetime e-cigarette use, and behavioral intentions.

Indoor Smoking Restrictions	E-Cigarette Use			Likely to Use E-Cigarettes in Next Year			
	Never	Lifetime		Not at all	Somewhat	Very	
	(*n* = 94)	(*n* = 169)	*p*-Value	(*n* = 77)	(*n* = 68)	(*n* = 120)	*p*-Value
Inside a home smoking traditional cigarettes should be ^*^			0.39				0.30
Never allowed	60% (56)	65% (109)		70% (54)	63% (42)	59% (71)	
Allowed at some times or places	22% (21)	24% (40)		17% (13)	27% (18)	25% (30)	
Always allowed	18% (17)	11% (19)		13% (10)	10% (7)	16% (19)	
Inside a home smoking e-cigarettes should be			0.04				<0.01
Never allowed	29% (27)	17% (29)		42% (32)	21% (14)	10% (12)	
Allowed at some times or places	37% (35)	35% (59)		39% (30)	42% (28)	30% (36)	
Always allowed	34% (32)	48% (80)		19% (15)	37% (25)	60% (72)	
At my home smoking cigarettes is			0.31				0.11
Never allowed	62% (58)	63% (106)		74% (57)	62% (42)	56% (67)	
Allowed at some times or places	12% (11)	17% (29)		8% (6)	16% (11)	19% (23)	
Always allowed	27% (25)	20% (34)		18% (14)	22% (15)	25% (30)	
At my home smoking e-cigarettes is			<0.01				<0.01
Never allowed	45% (42)	25% (42)		61% (47)	28% (19)	17% (20)	
Allowed at some times or places	18% (17)	26% (44)		16% (12)	28% (19)	25% (30)	
Always allowed	37% (35)	49% (83)		23% (18)	44% (30)	58% (70)	
At workplaces, smoking cigarettes should be ^*^			0.24				0.40
Never allowed	86% (81)	80% (136)		86% (66)	85% (58)	79% (95)	
Allowed at some times or places	13% (12)	18% (31)		14% (11)	15% (10)	18% (22)	
Always allowed	1% (1)	1% (2)		0% (0)	0% (0)	3% (3)	
At workplaces, smoking e-cigarettes should be			<0.01				<0.01
Never allowed	48% (45)	27% (46)		60% (46)	32% (22)	21% (25)	
Allowed at some times or places	35% (33)	44% (75)		30% (23)	46% (31)	45% (54)	
Always allowed	17% (16)	28% (48)		10% (8)	22% (15)	34% (41)	

^*^ Cigarettes sometimes allowed and always allowed combined for statistical analysis.

### 3.4. Associations between Support for E-Cigarette Bans, Lifetime Use, and Behavioral Intentions

No significant differences were found in support for indoor cigarette smoking bans, irrespective of e-cigarette use history (Table 4). That is, both lifetime e-cigarette users and never-users strongly supported complete bans on cigarette smoking in both home and work settings. However, this pattern differed with respect to e-cigarette restrictions. Complete e-cigarette bans in home settings were more likely to be supported by never-users of e-cigarettes, while no restrictions were more strongly endorsed by lifetime users. Compared to lifetime users, stronger personal bans on e-cigarette use at home were reported among never-users. Compared to e-cigarette never-users, participants with a lifetime history were more supportive of no restrictions and partial bans, respectively. In workplace settings, complete e-cigarette bans were strongly supported by never-users relative to lifetime users. Both partial bans and no restrictions were more supported by lifetime* versus* never-users.

No significant differences were found in support for indoor cigarette smoking bans, irrespective of behavioral intentions to use e-cigarettes within the next year (Table 4). That is, participants across the continuum of intentions to use e-cigarettes (from not at all to very likely) strongly supported complete cigarette smoking bans at homes and workplaces. However, this pattern varied with respect to e-cigarette restrictions. Complete e-cigarette bans in home settings were most supported by participants with no intentions to use e-cigarettes within the next year. In contrast, no restrictions were strongly endorsed among those with a high likelihood of future e-cigarette use. Compared to participants with at least some intention to use e-cigarettes, personal bans on e-cigarette use at home were strongest among those with no intentions to use e-cigarettes. No personal home restrictions were most supported when at least some intention to use e-cigarettes was present. Finally, at workplaces, complete e-cigarette bans were most supported by participants with no intentions to use the product, while no restrictions were most supported when strong intentions to use e-cigarettes were reported.

## 4. Discussion

This study was the first to characterize support for indoor e-cigarette bans among smokers and former smokers. We focused on home and workplace restrictions, and individual-level factors associated with support for bans (complete and partial). Endorsement of bans differed by product (*i.e.*, cigarettes* versus* e-cigarettes), and by age, marital status, income, and smoking status. Support for e-cigarette bans varied by risk perceptions (addiction potential and health risks), lifetime e-cigarette use history, and intentions to use e-cigarettes in the future. In contrast, support for complete and partial bans on cigarette smoking in homes and at work was consistently strong. Overall, findings from this study indicate that current and former smokers are relatively less supportive of restricting e-cigarette use in indoor settings when compared to cigarettes.

Support for indoor bans varied by product type. Respondents strongly supported restrictions on cigarette smoking in homes and workplaces. The majority of the sample indicated that cigarette smoking should be banned completely in home settings, including their own homes. Support for complete smoking bans in workplaces was even stronger. These findings are consistent with a recent Gallup poll [30], which reported that the majority of Americans are supportive of complete smoking bans in public places. NATS data [29] has also found strong support for cigarette smoking bans in public and private spaces in the U.S. [16,31]. In contrast, participants were significantly less supportive of complete or partial restrictions on e-cigarettes in either home or workplace settings. This is consistent with the increasing prevalence of e-cigarette use [19]. This is also a likely reflection of successful marketing strategies promoting e-cigarettes as safer alternatives to cigarette smoking [32] and limited data demonstrating the potential direct and secondhand exposure health risks [27]. We note, however, that a sizable minority of smokers and former smokers endorsed at least partial indoor bans on e-cigarette use.

We found significant differences in support for indoor e-cigarette bans by demographic factors. Younger, single, and lower income participants were less supportive of complete bans. Previous research has examined endorsement of smoking bans by sociodemographics for cigarettes, but not e-cigarettes. Decreased support for indoor cigarette smoking bans in public spaces has been observed among smokers, men, younger individuals and those with lower income and education [16,31]. The current study contributes to the extant literature by examining the association between sociodemographics and support for e-cigarette bans. Our findings also have implications for anti-e-cigarette public health campaigns, in that certain subgroups may be particularly vulnerable to e-cigarette marketing and use. Additional research is needed to understand the mechanisms underlying the lower levels of support for e-cigarette bans in these groups.

The U.S. Food and Drug Administration has issued statements regarding the potential danger of e-cigarettes [33] and is considering e-cigarette regulation. However, e-cigarettes are marketed as safe alternatives to cigarettes. We found consistent associations between e-cigarette risk perceptions and support for indoor bans. The strongest supporters of complete e-cigarette bans in home and work settings were participants who perceived e-cigarettes to be highly addictive; partial bans were most supported when e-cigarettes were perceived as somewhat addictive; and, no restrictions were strongly endorsed when perceived as non-addictive. Similar associations were found between support for indoor e-cigarette restrictions and health risk perceptions. That is, there was a dose response relationship between support for e-cigarette bans and perceptions of health risk. Importantly, only a minority of the sample were fully supportive of e-cigarette bans in homes and workplaces. This was in contrast to cigarettes, for which there was strong support for complete indoor bans. Previous research has not examined relationships between risk perceptions and e-cigarette bans. However, it is plausible that as evidence emerges regarding short or long-term health effects (or lack thereof), support for e-cigarette bans may change over time.

Lifetime e-cigarette use and future intentions to use e-cigarettes were inversely associated with support for e-cigarette bans. Those with a high likelihood of future e-cigarette use were also most likely to favor no restrictions. In contrast, complete e-cigarette bans were more likely to be supported by never-users. These findings are consistent with Wolfson *et al.* [18], who found that ever-smokers of cigarettes and/or e-cigarettes were less likely to support indoor restaurant bans of e-cigarettes than their counterparts.

## 5. Limitations

Findings should be considered in light of the limitations of this study. Participants were respondents to advertisements for a survey on cigarettes and e-cigarettes. This has implications for the variables assessed (e.g., lifetime e-cigarette use, risk perceptions, and intentions to use e-cigarettes) because they self-selected into the study and may not be representative of the general population. Other notable differences between our sample and those of other studies include the high proportion of racial/ethnic minorities, women, and low-income participants. We view the diversity of the sample as a strength, as these groups are underrepresented in the e-cigarette literature. Moreover, the pattern of findings was consistent across items, and with previous research [22]. In addition, the findings may not be representative of other geographic locations or treatment-seeking smokers. Finally, this study did not include nonsmokers; thus future research should also assess support for e-cigarette bans in this group.

## 6. Conclusions and Future Directions

Notwithstanding its limitations, this study makes an important contribution to the emerging literature by examining support of e-cigarette bans among former and current smokers, as well as correlates of bans support. This exploratory study found that relative to cigarettes, fewer participants favored complete e-cigarette smoking bans. However, support for e-cigarette bans varied by subgroups and individual-level variables. As debates surrounding e-cigarettes as harm reduction for smokers and the risks of secondhand exposure continue, public views regarding their use and possible restriction are important considerations. Findings from this study can be used to inform future research, public health and policy-related efforts.
